# Development of practice guidelines for hemodialysis in Egypt

**DOI:** 10.4103/0971-4065.73450

**Published:** 2010-10

**Authors:** A. M. A. Ahmed, Mohd. F. Allam, E. S. Habil, A. M. Metwally, N. A. Ibrahiem, M. Radwan, M. M. El-Gaafary, A. Afifi, M. A. Gadallah

**Affiliations:** National Research Center, Ain Shams University, Cairo, Egypt; 1 Department of Community, Environmental and Occupational Medicine, Faculty of Medicine, Ain Shams University, Cairo, Egypt; 2 Department of Nephrology, Faculty of Medicine, Ain Shams University, Cairo, Egypt

**Keywords:** Hemodialysis, Guidelines, Egypt

## Abstract

Although hemodialysis is the main modaility of treatment of end-stage renal disease, no practice guidelines are available in Egypt. Applying international guidelines for hemodialysis would not be suitable or feasible, because of different health system and lack of resources. The aim of this project was the development of evidence- and consensus-based clinical practice guidelines for hemodialysis in Egypt. The Egyptian guidelines were adopted from the standards developed by The College of Physicians and Surgeons of Alberta (Canada), The National Kidney Foundation (USA), The Clinical Standards Board for Scotland (Scotland), and The College of Physicians and Surgeons of Ontario (Canada). In addition, the guidelines published in Oxford Handbook of Dialysis were reviewed. Thereafter, a panel of Egyptian experts in the field of nephrology and hemodialysis was selected and invited to participate in this project. The Delphi technique was applied to build up the consensus among the experts on the formulated guidelines. The final version of the Egyptian Hemodialysis Practice Guidelines included five main sections; personnel, patient care practices, infection prevention and control, facility, and documentation/records. A consensus on practice guidelines for hemodialysis has been successfully produced and is supported by levels of evidence. The 12 Egyptian experts who participated in the Delphi technique and the reviewers assured the completeness and acceptability of the developed practice guidelines. Also, including experts from the university hospitals together with the Directorates of Cairo and Giza Health Affairs of the Egyptian Ministry of Health (MOH) avoided conflicts between clinical recommendations and feasible application in the MOH hemodialysis facilities.

## Introduction

End-stage renal disease (ESRD) is one of the main health problems in Egypt. Currently, hemodialysis represents the main mode for treatment of chronic kidney disease stage 5 (CKD5), previously called ESRD or chronic renal failure.[1]

In Egypt, the estimated annual incidence of ESRD is around 74 per million and the total prevalence of patients on dialysis is 264 per million.[2] Hemodialysis centers in Egypt exist in governmental, military, and university hospitals as well in the private sector. The average cost of the hemodialysis session ranges from US $16 in governmental hospitals to around US $32 in some private centers. The main hemodialysis regimen adopted in Egypt is three times per week. Most Egyptian centers are equipped with machines with controlled ultrafiltration and synthetic membranes. Many centers use bicarbonate buffer and high flux dialysers although they are not universally applied.[1] The hemodialysis centers, whether private or governmental, are under supervision by the Egyptian Ministry of Health (MOH). However, no Egyptian guidelines or approved guidelines to standardize the practice of hemodialysis are implemented in Egypt. Therefore, hemodialysis is not uniformly practiced across the different centers in Egypt. Adding to this, the practice of hemodialysis in some university centers; considered the highest level of care provision, showed no more than partial compliance with the international guidelines.[3]

There is sufficient evidence that implementation of quality management in the health sector will reduce the cost and provide higher quality. It is imperative to be understood that quality assessment and improvement is the way of the future in the healthcare industry.[4,5] Quality used to be defined objectively as compliance with, or adherence to standards. It is assumed that quality can be adequately, if not completely, measured once healthcare professionals define the standards of care under which they can comfortably practice. Acceptable compliance with standards is now the basis for granting healthcare organizations licensure/accreditation.[4]

In the United States, the National Kidney Foundation Dialysis Outcome Quality Initiative (NKF-DOQI) selected some aspects of the management of ESRD and developed, to date, 13 practice guidelines including vascular access for hemodialysis patients; treatment of anemia for patients with chronic renal failure; adequacy of hemodialysis; and adequacy of peritoneal dialysis.[6] Other organizations have established practice guidelines for the management of ESRD. For example, the Canadian Society of Nephrology guidelines selected some areas included in NKF-DOQI guidelines and added other not directly addressed by NKF-DOQI which is the management of patients with chronic renal failure before dialysis, particularly with respect to timing of initiation of dialysis.[7] The European Renal Association-European Dialysis and Transplant Association also added some guidelines in respect to water treatment and biocompatibility.[8]

The National Program of Accreditation in Egypt has established some standards for certain clinical areas in hospital services including surgery, anesthesia but has not yet established standards for hemodialysis.

The objective of the study was to develop practice guidelines for hemodialysis in Egypt and developing countries.

## Methods

### Research setting

The development of the Egyptian Haemodialysis Practice Guidelines entailed the participation of experts in Nephrology and Healthcare Quality Management from Cairo, Giza, and Minia governorates.

### Duration and time

The duration of the study was 12 months, from April 16, 2005 to April 15, 2006.

### Research methodology

**Figure 1:**
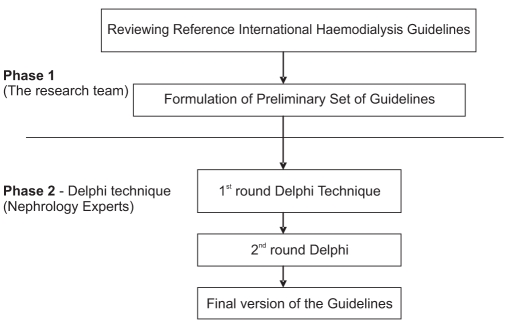
Steps of developing the hemodialysis guidelines in the current research

This study is considered one of the health system researches. The study was carried out in two phases [Figure 1]:

#### Phase I

In this phase, preliminary quality standards were formulated through:

a) Review of literature to collect the quality standards that have already been implemented in different countries including NKF-DOQI,[9] The Clinical Standards for Adult Renal Services in Scotland,[10] Clinical Practice Parameters and Facility Standards for Hemodialysis by the College of Physicians and Surgeons of Ontario,[11] and Guidelines published in Oxford Handbook of Dialysis.[12]

The above-mentioned sets of standards combine both evidence-based standards and other opinion-based standards. Example of evidence-based guideline is the regular measurement of the delivered dose of hemodialysis and the method of its measurement using *Kt/V*. Other guidelines are opinion based such as examination of the site of the fistula for signs of infection. The level of evidence was examined through systemic reviewing of related articles keeping the consensus opinion for practices having limited researches.

b) A preliminary version of the Egyptian practice guidelines for hemodialysis was formulated with the contribution of an expert of nephrology.

#### Phase II

The Delphi technique was applied to build up a consensus among the experts on the preliminary version of the formulated practice guidelines.[13] This consensus building technique among the Egyptian experts has the objective to ensure applicability of the formulated guidelines in the Egyptian practice. The Delphi technique is a structured interactive method involving repetitive administration of anonymous questionnaires, usually across two or three postal rounds. Face-to-face meetings are not usually a feature. The main stages include: identifying a research problem, developing questionnaire statements to rate, selecting appropriate panellists, conducting anonymous iterative postal questionnaire rounds, feeding back results (statistical, qualitative, or both) between rounds, and summarizing and feeding back the findings. The approach enables a large group to be consulted from a geographically dispersed population.

The technique was applied to 12 experts from different public universities, MOH, and other health care facilities, including private sector.

The selection of the experts was done after a meeting attended by the Project Team, Professor of Nephrology and representative of the Department of Nephrology in Giza Governorate (MOH). The selection of the experts was based on experience, high educational background, and to cover diverse geographical areas. The objective of this meeting was to ensure the selection and participation of nephrologists from different health sectors in Egypt.

The first version of the formulated practice guidelines was delivered to the Egyptian experts after explaining the aim and methodology of the study (first round). After 2–3 weeks, the responses were collected and analysed. Thereafter, the areas of disagreement were identified and highlighted. The second version of the guidelines was delivered to the same experts of the first round after clearing up the areas of disagreement and considering the different recommendations of the panel experts regarding them (second round). After 2–3 weeks, the responses were collected and analysed to formulate the third version of the practice guidelines. Thereafter, a meeting was organized and four experts were invited to review the third version in order to reach a final format for the practice guidelines. The four experts included two who previously participated in the first and the second rounds of the Delphi technique and two new experts; the director of the hemodialysis center in a university hospital and the supervisor on hemodialysis centers in Cairo. After the meeting, the final version of the Egyptian Hemodialysis Practice Guidelines was formulated. The final version was reviewed by a Professor of Nephrology in Ain Shams University. Thereafter, the reviewed version was delivered to Professor of Nephrology in Cairo University and Chair of the Clinical Practice Guidelines Committee—The International Society of Nephrology for final revision and approval.

## Results

The final version of the Egyptian Hemodialysis Practice Guidelines included five main sections; (1) personnel, (2) patient care practices, (3) infection prevention and control, (4) facility, and (5) documentation/records (Appendix).

These formulated guidelines are prepared in nine pages. The personnel section included the staffing pattern and their qualifications. Patient care practices included the writing order for hemodialysis, pre-dialysis preparation, dialysis process, and care between dialysis sessions. Infection prevention and control section covered the different preventive measures for the patients, medical staff, reprocessing, sterilization, and disinfection for equipments and surfaces as well the housekeeping and waste management measures. The facility guidelines included the physical standards as well the administrative standards. The last section of the guidelines consisted of the documentation and records maintaining.

## Discussion

Healthcare facilities are seeking nowadays to develop practice guidelines for the sake of improving healthcare services. In the healthcare sector in Egypt, trials for establishing guidelines have been lead by the MOH.[14]

Although hemodialysis is often used for treatment of ESRD, no practice guidelines are available in Egypt. Worldwide, international organizations set some guidelines like the National Kidney Foundation, which published guidelines concerning the adequacy of hemodialysis, adequacy of peritoneal dialysis, treatment of anemia in chronic renal failure patients, and vascular access.[9] Other individual institutions developed their own guidelines for hemodialysis, which differ according to the accreditation requirements, or the institution administration policy. Almost all the guidelines adopted followed the international guidelines for areas related to hemodialysis such as the infection control and guidelines published by the National Kidney Foundation.

Applying international guidelines for hemodialysis in Egypt would not be suitable or feasible, because of different health system and lack of resources. The main aim of this project was the development of evidence- and consensus-based clinical practice guidelines for hemodialysis in Egypt.

Clinical practice guidelines often grade the “strength” of their recommendations according to the robustness of the supporting research evidence.[15]

Accordingly, we reviewed five practice guidelines from four developed countries to formulate the first set of the Egyptian hemodialysis practice guidelines. Thus, the first version of the Egyptian guidelines was not identical to any of the reviewed practice guidelines, and rather included all controversial issues.

Using the Delphi technique, an exercise was undertaken to ascertain whether a consensus on general protocol for hemodialysis practice could be reached among Egyptian experts. The main problem at this stage was the selection of the Egyptian experts. Currently, Egypt has 17 medical schools together with specialized medical institutes. Selection of the Egyptian experts was a tiresome job. We decided to include consultants from all health sectors in Egypt rather than including only university professors or specialized institutes consultants. Healthcare in Egypt is offered through different sectors (e.g., general public hospitals, specialized public hospitals, health insurance organization, university hospitals, specialized institutes hospitals, police hospitals, and private hospitals), thus it was recommendable to include at least an expert from every sector. We also included an expert from the Directorate of Giza Health Affairs, to be sure that the practice guidelines will be acceptable and recommendable by stakeholders in the Egyptian MOH.

In general, a panel of 12 experts to build a consensus on national practice guidelines is more or less enough. Previously, the Surviving Sepsis Campaign included critical care and infectious disease experts representing 11 international organizations to develop management guidelines for severe sepsis and septic shock.[16]

One round was not enough to develop the Egyptian hemodialysis guidelines, but a second round was conducted on conflicting issues and detailed explanation was given regarding these issues to all experts, including the different American and European recommendations.

The developed practice guidelines after the two rounds were reviewed in a meeting, which included two university professors and two experts from the Directorates of Cairo and Giza Health Affairs. This meeting aimed to ensure the completeness of and acceptability to the practice guidelines, especially that two of the four participants were not included in the Delphi technique. These two new participants were the director of the hemodialysis unit in a university hospital and the supervisor on hemodialysis centers in Cairo governorate, to avoid conflicts between clinical recommendations and feasible application in the MOH hemodialysis facilities.

An example of a conflicting issue was the frequency of measuring *Kt/V*. Although all international guidelines recommended its measurement every month, our guidelines recommended its measurement, after the meeting, every 3 months, because of its high cost. We referred in this case to the practice guidelines published in Oxford Handbook of Dialysis (2004).[12]

The President of the National Kidney Foundation, a branch of the Egyptian Society of Nephrology, reviewed the final developed practice guidelines. Previously, the Egyptian Society of Nephrology and Transplantation developed management guidelines for hemodialysis, especially concerning the personnel item.

The revised guidelines were reviewed again by the Chair of the Clinical Practice Guidelines Committee (The International Society of Nephrology), who is also the Past-President of the Egyptian Society of Nephrology and Professor of Nephrology at Cairo University, to ensure compliance with the international guidelines and appropriateness for application in Egypt.

According to basic concept of evidence based-medicine, half of all we are taught will in 10 years show to be wrong. Thus, the developed Egyptian Hemodialysis Practice Guidelines should be reviewed and updated regularly by the MOH and the Egyptian Society of Nephrology and Transplantation.

The process of reviewing the published guidelines and quality standards in hemodialysis units tried to include multiple sources to avoid monopoly as well to broaden the options. However, one aspect that has been curiously looked for is the nonclinical administrative and facility quality standards applicable in the hemodialysis centers. This aspect is often unclear or improper in the common organizations delivering hemodialysis in Egypt. Although few published guidelines were used as a basis for this work, yet most of the published guidelines in this field agree with their recommendations and those used in this research are not by any means superior but more or less covering some missing aspects in the Egyptian practice such as the administrative and facility organization.

The present study is an initial step in preparing a set of Guidelines for Hemodialysis that are applicable for the Egyptian practice to cover most of the areas in any hemodialysis center including the personnel and practice. A complementary part for this study is to validate the selected standards by checking the baseline compliance of these guidelines in some hemodialysis centers and hence gives an idea on the gaps and the difference in practices which will evidently determine the further integration of these standards in the regular practice. It will highlight the necessary training areas as well the baseline data after which monitoring will be carried out.

### Recommendations

These formulated guidelines were communicated to the quality directorate in the MOH in Egypt. However, the implementation of such guidelines is, till the date of submitting this manuscript, in its first evolutionary steps and depending mainly on individual efforts. Accordingly, the following is recommended to ensure wide application of such standards:

1. The formulated practice guidelines for hemodialysis should be distributed to all hemodialysis facilities in Egypt. It will be advisable to print the Egyptian hemodialysis guidelines as a small handbook, which could be delivered to all healthcare workers working in hemodialysis facilities.
2. Organize 1-day workshops for hemodialysis consultants and directors of hemodialysis facilities to orient them with all items of the developed practice guidelines. Attendants should be from both public and private sectors.
3. The developed practice guidelines should be reviewed and updated, if needed, yearly. The Egyptian Society of Nephrology and Transplantation could take care of this scientific task.

## APPENDIX

### Egyptian standards and guidelines for hemodialysis

#### Introduction

End-stage renal disease (ESRD) is one of the main health problems in Egypt. Currently, hemodialysis represents the main mode for treatment of chronic kidney disease stage 5 (CKD5), previously called ESRD or chronic renal failure.

It is to be noted that health care service in hemodialysis facility extends to include infection control, patients’ data recording, and other medical services. All together determine the final outcome of hemodialysis.

This is called for developing the Egyptian Standards and Guidelines for the basic hemodialysis practice in different facilities.

The present Egyptian guidelines were adopted from the standards developed by The National Kidney Foundation (USA), The Clinical Standards Board for Scotland (Scotland), and The Collage of Physicians and Surgeons of Ontario (Canada). Also, we reviewed the guidelines published in Oxford Handbook of Dialysis (2004). Thereafter, a panel of Egyptian experts in the field Nephrology and Hemodialysis was selected and invited to participate in this project. The Delphi technique was applied to build up the consensus among the experts on the formulated guidelines.

Nevertheless, the set of standards and guidelines can be regarded as general protocol for hemodialysis practice. Specific guidelines and protocols can be developed and adopted for any specific problem.

These standards are the yield of a collaborative project between the National Training Institute (Egyptian Ministry of Health and Population) and the Eastern Mediterranean Regional Office (World Health Organization).

The Egyptian Standards and Guidelines for Hemodialysis Project was funded by a grant from EMRO/WHO (RPC TSA 05/8).

#### Standards and guidelines

- **Personnel**
  1. Consultant:
    1.1. Physician with valid license in Egypt
    1.2. Registered as Consultant of Nephrology in the Egyptian Society of Nephrology and/or the Egyptian Medical Syndicate
    1.3 Current certificate in basic Cardiac Life Support
  2. Medical Director:
    2.1. Physician with valid license in Egypt
    2.2. Has a Master Degree or Diploma in Nephrology, Internal Medicine, or Pediatrics
    2.3. Has completed training in hemodialysis in a referral center
    2.4. Experience in hemodialysis for at least 5 years
    2.5. Current certificate in basic Cardiac Life Support
  3. Hemodialysis Physician:
    3.1. Physician with valid license in Egypt
    3.2. Has completed training in Internal Medicine or Pediatrics for at least 1 year
    3.3. Has completed training in hemodialysis in a referral center for at least 6 months
    3.4. Current certificate in basic Cardiac Life Support
  4. Nursing supervisor:
    4.1. Registered nurse
    4.2. Formal training in hemodialysis in a referral center
    4.3. Experience in hemodialysis for at least 3 years
    4.4. Current certificate in basic Cardiac Life Support
  5. Dialysis Nurse:
    5.1. Registered nurse
    5.2. Formal training in hemodialysis in a referral center
    5.3. Current certificate in basic cardiac life support
  6. taffing:
    Hemodialysis physician and Nurse supervisor for every eight patients in every session
    Nurse for every three patients in every session
- **Patient Care Practices**
  1. **Writing orders for hemodialysis**
    1. Hemodialysis delivery system
    2. Hemodialyser
    3. Blood flow rate
    4. Dialysate composition
    Sodium
    Potassium
    Calcium
    Glucose
    Buffer base
    5. Dialysate flow rate
    6. Frequency and duration of treatment
    7. Ideal or dry weight
    8. Amount of weight to remove
    9. Type and amount of fluid to support blood pressure
    10. Heparin doses in details
    11. Laboratory tests
    Initial
    Monthly
    Quarterly
    Biyearly
    Yearly
    12. Nutritional management according to dietitian’s instructions
    13. Intradialytic medications
    14. Method of monitoring adequacy of dialysis: urea reduction ratio or *Kt/V* every 3 months
    15. Any special instruction like drug allergy
    Timing: At start then reviewed every month
  2. **Predialysis preparation**
    - Patient assessment:
      – Temperature
      – Pulse
      – Blood pressure
      – Weight
      – Vascular access (Inspection and palpation for signs of infection and checking arterial bruit)
      – Recent medical history and general physical examination
      – Review previous lab results
    - Machine parameters assessment:
      – Type of dialyser
      – Delivery system
      – Bath concentration
      – Safety checks
  3. **Dialysis**
    - Initiation of dialysis:
      1. Gather all supplies before initiation of dialysis
        1. Clamps for blood lines
        2. Gloves
        3. Gauze pads
        4. Tape
        5. Alcohol wipes and/or betadine swabs
        6. Tubes for predialysis laboratory tests
        7. Drapes or barrier
        8. Needles and syringes
        9. Heparin
        10. Lignocaine
        11. Fistula needles or shunt adaptor
        12. Treatment record
        13. Physician orders
      2. Wash hands and wear gloves
      3. Prepare access using betadine scrub OR Wash the limb with soap and water, and then clean with alcohol-based solution for at least 3 min. Thereafter, the vessel should not be palpated
      4. Anesthetize the skin using 0.5% lignocaine (0.1–0.5 mL s.c.): Optional for selected patients
      5. Canulation technique
        i) Insert the arterial needle first
        ii) Insert the venous needle second
        iii) Use the button-hole approach (less rate of aneurysm than the rope ladder technique)
        If canulation is unsuccessful three times, repeated attempts should be avoided and vascular consultation is warranted. If the site becomes swollen the area should be avoided until the swelling/bruising has gone.
    - Monitoring
      Frequency
      Every Author but may be more frequently
      Vital signs (pulse and blood pressure)
      Subjective symptoms and signs
      Arterial and venous pressure monitoring
      Heparin pump, mL delivered
      Blood pump speed, mL/min
      Arterial and venous pressure limits
      Color of blood and dialyser
      Blood lines and circuit integrity
      Temperature: Pre- and postdialysis and when needed
    - Termination of dialysis
      1. Gather all supplies
        2.1. Wash hands
        2.2. Wear gloves
      2. Start and termination:
        1. Reset the arterial and venous monitor gauges to wider limits
        2. Stop the blood pump
        3. Clamp the arterial needle line
        4. Clamp the arterial machine lines
        5. Turn off the negative pressure (ultrafiltration control)
        6. Observe the venous line to the patient, ensuring there are no air bubbles
        7. Disconnect the arterial line from the arterial needle and hold the arterial line above the level of dialyser
        8. Turn the blood pump to 100 mL/min and unclamp the arterial line, to return the blood with the saline infusion line
        9. Clamp the arterial line and unclamp the saline line
        10. Reinfuse according to the standard protocol
  4. **Postdialysis**
    - Charting the following:
      1. Vital signs, including standing BP
      2. Weight
      3. Weight loss
      4. Total fluid received
      5. Total anticoagulant received
      6. Saline or other colloid or crystalloid required to support BP
      7. Complications
    - clean the machine and disinfection according to a standard protoccol
  5. **Between dialysis**
    - Laboratory Testing
      i) Every 1 month
      1. CBC, including platelet count.
      ii) Every 3 months
      1. BUN pre- and postdialysis to calculate URR, Kt/V (preferably every month)
      2. Creatinine
      3. Electrolytes (Na, K, and Cl)
      4. Calcium and phosphorus
      5. Glucose (fasting and post prandial)
      6. Bilirubin
      7. SGOT and SGPT
      8. Alkaline phosphatase
      9. Albumin
      10. HBsAg
      11. HCV antibody
      12. HIV antibody
      13. C-reactive protein
      iii) Every 6 months
      1. Iron and Iron-binding capacity
      2. Ferritin
      3. Parathyroid hormone
      iv) Every 12 months
      1. Chest X-ray
      2. Electrocardiogram
    - Nutrition education by dietitian or referral to primary health care unit nutritionist
    - Social Service through the social worker of the hemodialysis unit or the Primary Health Care Unit
    - The adequacy of hemodialysis is assessed by measuring the urea reduction ratio (at least ≥65) or Kt/V (at least 1.2) The facility should record the number of persons with urea reduction ratio < 65% or Kt/V < 1.2 over the total number of patients dialyzed
- **Infection Prevention and Control**
  1. Vaccination of Patients
    All patients should be vaccinated against Hepatitis B, according to the guidelines for vaccinating of a patient with end stage renal disease.
  2. Infectious Disease/Occupational Health and Immunization
    1. Immunization should be offered to staff if immune status is unknown or inadequate. Immunization standards for staff include:
      - Hepatitis B vaccine—highly recommended for all personnel at risk of potentially harmful contact with blood and body fluids.
      - Tetanus toxoid—recommended at 10-year intervals.
    2. All personnel shall understand and adhere to standard (universal) blood and body fluid precautions.
      Those precautions include:
      i) The proper use of personal protective devices such as gowns, gloves, visors, and masks.
      ii) The proper use and disposal of sharp devices.
      iii) An approved method for the disposal of blood and body fluid spills.
      iv) Appropriate hand washing practices.
      v) Appropriate disinfection of hemodialysis equipment including internal and external surfaces.
  3. General infection prevention measures
    1. Adequate hand washing sinks shall be appropriately located throughout the facility. A sufficient supply of cloth or disposable towels shall be available so that a fresh towel can be used after each hand washing. Common towels are prohibited
    2. Hands shall be washed between patients, after removal of gloves, and after contact with any contaminated objects. Washing hands with gauze and alcohol between patients in cases of increase work load may be accepted although proper hand washing with water and soap is recommended in all cases
    3. The use of multidose vials is strongly discouraged. If they are used, care shall be taken to not contaminate the contents of the vial. The rubber diaphragm shall be wiped with alcohol, and a clean needle and clean syringe shall be used each time the vial is entered. The date the vial is first used shall be recorded on the vial. The opened vial should be discarded within a period recommended by the manufacturer or within one month if longer storage (than the manufacturer’s recommendation) can be shown to be safe
    4. Drugs shall never be delivered to more than one patient or IV system attached to the patient from a common syringe or IV bag
    5. Smoking shall not be permitted in any area of the facility
    6. Personnel and patient must not eat or drink in any area where direct care is provided
    7. Linen, bed, and pillow covers shall be changed between patients. Beds and stretchers shall be wiped down between patients with a quaternary ammonia compound in accordance with manufacturer’s instructions
    8. Patient care items such as K-basins, thermometers, etc., shall not be used between patients unless reprocessed according to the manufacturer’s guidelines. Other items, such as cups, should be disposable.
    9. No animals should be allowed in the facility
    10. The facility shall effectively be protected against the entrance of insects, animals or the elements by selfclosing doors, closed windows, screens, controlled air currents, or other effective means
    11. There shall be a designated person responsible for the maintenance and enforcement of infection control and occupational health standards in the facility who shall liaise with the infectious disease consultant for the facility
    12. There should be a written policy for managing patients with blood borne infections (HIV—HCV—HBV). This policy should determine if a separated labeled machine is used.
  4. Reprocessing, sterilization, and disinfection
    1. Adequate sterilization equipment shall be available and in working order (oven and autoclave)
    2. The guidelines for sterilization should be followed as in the National Guidelines for Infection Control
    3. Clean and soiled supplies shall be properly segregated and physically separated at all times
    4. There shall be a designated area for soiled supplies. This area shall be physically separated from patient care areas and from areas housing clean and sterile supplies
    5. The soiled area should have:
    - Adequate counter space to receive soiled supplies
    - A double utility sink to rinse and clean soiled items
    - A flushing device for the disposal of body fluid wastes
    - An adequate facility to hand wash (the dirty utility sink is not appropriate)
    6. Personnel working in the soiled area shall have proper protective apparel for their personal protection shall be properly trained, and should have hepatitis B immunization
    7. The clean area shall have adequate counter space for receiving washed equipment for storage or wrapping
    8. There should be written policies and procedures for the operation and maintenance of the sterilizers
    9. Routine preventive maintenance shall be performed on the sterilizer. This shall be documented
    10. There shall be a method to check the sterilization parameters of the equipment (i.e., temperature reached, time, etc.)
    11. There shall be appropriate monitoring of the sterilizers with biological monitors and a recall method for sterilized equipment (i.e., date and batch number clearly labeled on the package)
    12. Personnel operating the sterilizers shall be properly trained
    13. An approved method of sterilization shall be used
    14. Outside shipping cartons shall not be kept in the clean supply area. Deboxing of the cartons shall not be carried out in the clean area or in the patient care areas
  5. Housekeeping and Waste Management
    1. The location shall be kept neat, clean, and free of waste material
    2. Handling of waste material shall comply with the National Guidelines for Infection Control
    3. Housekeeping personnel shall be trained for the specific requirements of a health care facility and shall maintain an established housekeeping routine (schedule)
    4. Personnel shall adhere to a written protocol for cleaning each patient care area:
    1. Between patients
    2. At the end of the day
    3. Weekly
    4. Monthly
    5. Dry-mopping shall not be used
    6. Provisions shall be made for proper laundering of linen and washable goods
    7. Soiled linen shall be placed in containers and handled as little as possible and stored away from clean supplies
    8. Protective garments (e.g., leather gowns and leather gloves) should be worn to sort soiled linen if necessary
    9. All patient care linen shall be removed from the care area after each use
    10. Clean linen shall be covered and stored away from soiled linen
    11. Garbage shall be collected, contained, stored, and disposed so as to prevent disease transmission
    12. Sharps shall be disposed in clearly labeled punctureresistant containers, and transported and disposed of according to the local regulations. Leak-proof containers shall be used for disposal of all used hemodialysis supplies
    13. Sharps containers shall not be overfilled, and one container shall never be emptied into another
    14. Walls and floors in dialysis areas shall be washable
- **Facility**
  1. Administration standards
    An organizational chart is recommended and should be updated as necessary and be available to all personnel The duties and responsibilities of all personnel in the facility should be outlined in current written job descriptions There shall be adequate space for administrative functions so as not to interfere with clinical care and support areas
  2. General/Physical Standards
    2.1 The facility shall comply with all applicable building code and fire regulations
    2.2 The facility should be accessible to disabled persons.
    2.3 There shall be easy access by an ambulance and stretcher for transfer of emergency cases to the hospital
    2.4 The design of the facility shall provide for separate administration and patient waiting areas; clean utility, dirty utility and nonsterile storage; equipment storage; patient and staff lavatories; and water treatment as required
    2.5 Sterile and nonsterile areas shall be clearly demarcated
    2.6 The facility’s doors and corridors shall be wide enough for stretcher access
    2.7 There should be a sufficient number of reception and waiting areas to accommodate patients, relatives, and escorts
    2.8 Emergency lighting source (battery-operated or emergency power source) shall be available in all patient waiting areas and washrooms as well as in patient care areas unless natural light is available
    2.9 Fire extinguishers shall be available according to local standards
    2.10 There shall be a clean area to permit beverage preparation for patients
  3. Nursing station
    3.1 The nursing station shall have a clear view of the entire treatment area
    3.2 There shall be adequate storage for:
    - Patient records
    - Pharmaceutical supplies
    - Office supplies
    3.3 There shall be an adequate work area for writing notes
  4. Dialysis treatment area
    4.1 Each dialysis chair or bed shall occupy a minimum of six gross square meters
    4.2 The dialysis treatment area shall be free of extraneous materials such as boxes and supplies
    4.3 There shall be adequate sinks for implementing universal precautions
    4.4 There shall be eyewash stations available
- **Documentation/Records**
  There shall be an appropriate administrative structure to provide for the documentation, storage, and retrieval of all necessary patient information.
  1. Employee Records
    1.1 Appropriate personnel records shall be maintained in confidential files and should include:
    - A completed application form.
    - A record of having been oriented to the facility and of having received education in his/her assigned duties.
    - Evidence of required credentials (e.g., RN).
    - Performance evaluations.
    - Evidence of required certifications (e.g., cardiopulmonary resuscitation)
    - Vaccination and immune status records
  2. Medical Records
    NOTE: The clinical record is the property of the facility and the responsibility of the Medical Director
    2.1 The facility shall maintain a log book which contains the name of the patient and date of procedure
    2.2 Patients undergoing dialysis shall have a clinical record that originates with the initial visit and includes ongoing clinical record of the patient
    2.3 The clinical record shall follow a uniform format within the facility and be accurate, complete, and legible.
    This shall include:
    - Patient’s name
    - Home address
    - Second unique identifier (e.g., date of birth)
    2.4 Patients and equipment shall be assessed and documented before, during, and after dialysis
    2.5 The clinical record shall contain the following:
    i) ConsentConsent for the procedure signed by the patient and witnessed.
    ii) ConsentAssessment record which includes:
    - A medical history and problem list that is prepared or reviewed by the facility’s nephrologist
    - A dialysis prescription that includes dry weight
    - A list of current medications
    - Blood pressure
    - Allergies/adverse reactions
    - Results/reports of requested examinations, tests, consultations, and treatments (as indicated) including, at a minimum, reports of the most recent ECG, chest X-ray, HB, VRE, MRSA, and blood work
    - Name and contact information of referring physician and referring dialysis unit
    - Names of personnel performing assessments or procedures
    2.6 All reports containing interpretations shall indicate authorship and whether or not the author has verified the contents of the report (A signature means that the contents have been verified).
    2.7 Access to the clinical record shall comply with written policies ensuring confidentiality and determining those who may have access to the records
  3. Storage and Retention of Records
    3.1 Medical records and facility logs shall be maintained according to a written policy and the time frame after death or transfer should be determined
